# Galaxy-M: a Galaxy workflow for processing and analyzing direct infusion and liquid chromatography mass spectrometry-based metabolomics data

**DOI:** 10.1186/s13742-016-0115-8

**Published:** 2016-02-23

**Authors:** Robert L. Davidson, Ralf J. M. Weber, Haoyu Liu, Archana Sharma-Oates, Mark R. Viant

**Affiliations:** GigaScience, BGI-Hong Kong Co. Ltd, Tai Po Industrial Estate, 16 Dai Fu Street, Tai Po, NT Hong Kong; School of Biosciences, University of Birmingham, Birmingham, B15 2TT UK

**Keywords:** Metabolomics, Lipidomics, Workflow, Pipeline, Liquid chromatography mass spectrometry, LC-MS, Fourier transform ion cyclotron resonance, FT-ICR, Galaxy project, Reproducibility

## Abstract

**Background:**

Metabolomics is increasingly recognized as an invaluable tool in the biological, medical and environmental sciences yet lags behind the methodological maturity of other omics fields. To achieve its full potential, including the integration of multiple omics modalities, the accessibility, standardization and reproducibility of computational metabolomics tools must be improved significantly.

**Results:**

Here we present our end-to-end mass spectrometry metabolomics workflow in the widely used platform, Galaxy. Named Galaxy-M, our workflow has been developed for both direct infusion mass spectrometry (DIMS) and liquid chromatography mass spectrometry (LC-MS) metabolomics. The range of tools presented spans from processing of raw data, e.g. peak picking and alignment, through data cleansing, e.g. missing value imputation, to preparation for statistical analysis, e.g. normalization and scaling, and principal components analysis (PCA) with associated statistical evaluation. We demonstrate the ease of using these Galaxy workflows via the analysis of DIMS and LC-MS datasets, and provide PCA scores and associated statistics to help other users to ensure that they can accurately repeat the processing and analysis of these two datasets. Galaxy and data are all provided pre-installed in a virtual machine (VM) that can be downloaded from the GigaDB repository. Additionally, source code, executables and installation instructions are available from GitHub.

**Conclusions:**

The Galaxy platform has enabled us to produce an easily accessible and reproducible computational metabolomics workflow. More tools could be added by the community to expand its functionality. We recommend that Galaxy-M workflow files are included within the supplementary information of publications, enabling metabolomics studies to achieve greater reproducibility.

**Electronic supplementary material:**

The online version of this article (doi:10.1186/s13742-016-0115-8) contains supplementary material, which is available to authorized users.

## Findings

### Introduction

Omics studies, such as genomics and metabolomics, are transforming our mechanistic understandings of biological processes from human ageing and disease to environment toxicology and ecology [1–4]. Metabolomics has been described as the ‘real-world endpoint’ of omics research and acknowledged as having the potential to bridge the gap between genotype and phenotype [5]. While genomics research uses established analytical technologies and standardized data analysis platforms, metabolomics is less developed both analytically and computationally.

Metabolomics research typically includes workflows from data collection through signal processing, statistical analysis and ultimately to the annotation or identification of metabolites. The technologies involved are not fully mature, with researchers using multiple analytical platforms (e.g. liquid chromatography mass spectrometry (LC-MS), gas chromatography (GC)-MS, direct infusion (DI)MS and nuclear magnetic resonance (NMR) spectroscopy), yielding multiple data formats which can then be processed and analyzed using a plethora of tools (e.g. XCMS, mzMatch, mzMine, PLS-Toolbox) and the metabolites identified (e.g. Camera, PUTMEDID and MI-Pack software) using compound reference databases (e.g. KEGG, HMDB and LIPID MAPS), and finally deposited in data repositories (e.g. MetaboLights) [6–8]. Although several high profile standardized analytical protocols have been published, e.g. for non-targeted LC-MS [7], DIMS [9–11] and NMR [12], there are only a limited number of metabolomics-specific computational workflows available that incorporate the software tools and databases introduced above and that do not require programming expertise [13]. The implementation of computational platforms to conduct accessible, reproducible and transparent metabolomics research is an urgent need for the community. The establishment of such approaches will further advance the robustness, standardization, deployability and impact of metabolomics research, increasing the data quality and eventually facilitating its integration with other omics domains.

There are many workflow platforms that have been implemented successfully across a variety of scientific fields [14–16]. Recently, several in-house as well as community-based open source workflow platforms (e.g. Taverna [17] and Galaxy [18]) have been developed and implemented. Galaxy has emerged as one of the leading open source workflow platforms for next generation sequencing (NGS) data analysis, with many standard processing tools accessible from its web-based user interface (e.g. by June 2014, the number of registered Galaxy main users reached approximately 55,000 [19]). This has enabled biologists without programming skills to construct and execute NGS data analyses. Galaxy workflows have also begun to emerge in proteomics research [20].

### Purpose of this work

This article has three primary objectives: first, to help to introduce the wider metabolomics community, from bioinformaticians to practising metabolomicists, to the benefits of Galaxy workflows. Second, to promote some understanding of Galaxy workflows beyond simply how they are accessed and used by a practitioner. Such an awareness of the underlying methods and their assumptions is important for avoiding any misuse of Galaxy workflows. Finally, to bring a set of non-targeted DIMS and LC-MS based metabolomics processing and analysis tools into the Galaxy workflow platform. With this we aim to strengthen the move towards standardized, reproducible, transparent and shareable workflows in metabolomics while providing a much more intuitive interface for researchers without programming experience and ultimately providing a platform that can integrate this omics approach with the many others that already exist in the Galaxy environment (e.g. genomics and proteomics).

### Implementation

**Fig. 1:**
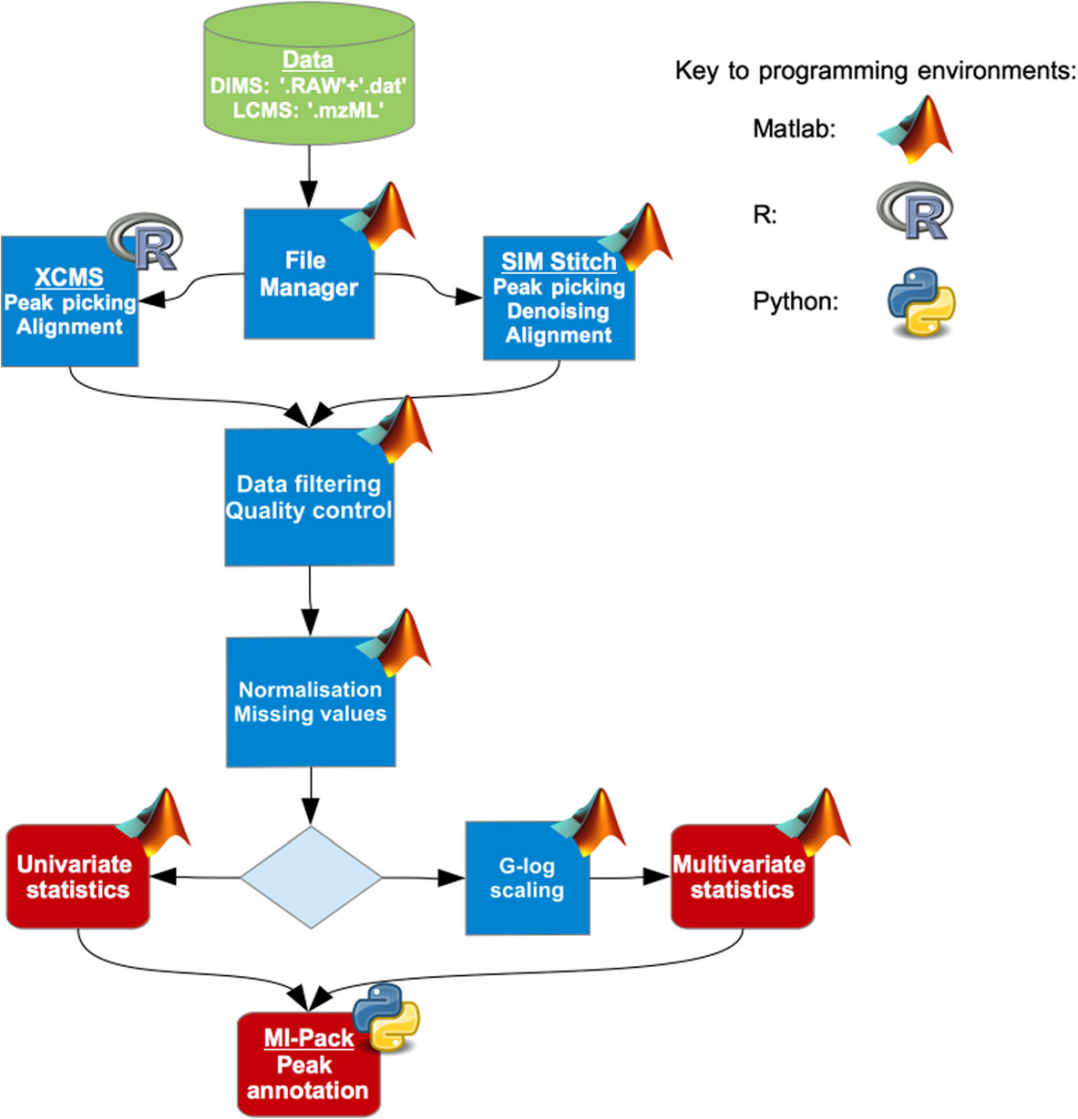
Overview of Galaxy-M metabolomics workflow for DIMS and LC-MS data. Processing of raw data is performed either using SIM-stitch for DIMS data or XCMS for LC-MS data [9, 23]. Metabolite annotation is performed using MI-Pack [10]. Logos denote programming environments for each stage of the data processing. Note that no univariate statistics tools are included in the current implementation

We have implemented Galaxy workflows for two widely used non-targeted metabolomic modalities, DIMS using an LTQ FT Ultra Fourier transform ion cyclotron resonance (FT-ICR) mass spectrometer (Thermo Scientific, Waltham, USA), and LC-MS using a Prominence LC (Shimadzu, Tokyo, Japan) coupled to an LTQ Orbitrap Velos (Thermo Scientific, Waltham, USA) mass spectrometer. The DIMS workflow was developed within our own laboratory over the past few years [9–11, 21, 22] and includes multiple steps to process raw data files, to prepare the data matrix (X), to conduct statistical analysis, and finally to annotate the metabolites (Fig. 1). This mass spectrometric method comprises the collection of multiple adjacent selected ion monitoring (SIM) windows that are ‘stitched’ together computationally, hence the name ‘SIM-stitching’, providing increased metabolome coverage, very high mass accuracy, and at 2 min 15 s analysis time per sample is conducive for high-throughput metabolomics. The specific LC-MS workflow implemented here has not been used previously in published work; it has been included as a representative series of processing steps to further demonstrate the ease of working in the Galaxy environment. The LC-MS data is processed using XCMS [23], including feature detection, retention time correction and alignment (Fig. 1). Together, the DIMS and LC-MS workflows represent core requirements for a generic ‘metabolomics analysis’, from start to end. We provide the workflows and all analyses in a fully operational Galaxy installation within a virtual machine (VM) that is stored in the GigaDB repository [24]. Our combined system makes use of Python, R and Matlab programming languages, this complex environment showing another benefit of the unified interface provided by Galaxy. The code in all cases is provided as ‘open source’ via GigaDB and GitHub, but in the case of Matlab a license is also required to run these tools direct from source. An alternative is provided by inclusion of compiled, standalone versions of each Matlab-based tool; thus the whole workflow can be run without the need for purchased, proprietary licenses.

### Data handling

Data is accepted in its raw state. In the case of DIMS, this comprises a single .RAW format file or a .RAW format file together with a large number of .dat files (i.e., transient data) that represent the mass spectrometric data from within each SIM window; for LC-MS there will be a single .mzML file for each sample. For most metabolomics studies this represents a large number of files and a considerable amount of data to be uploaded to the Galaxy server (i.e. multiple Gigabytes), to then be held in the history and combined by subsequent tools. Our implementation therefore assumes that the user will store their data on a file system that is directly accessible by the Galaxy system (i.e. no Galaxy upload required). The initial tools merely ask for the location of a directory containing these raw data files. This does not make full use of Galaxy’s advanced functions (e.g. Shared Data Libraries) and on a production system could even pose a security risk, but for the inexperienced Galaxy user, we feel that this use of file paths is the easiest method.

The DIMS workflow includes a tool named ‘File List Manager’, which takes as input the directory containing the .RAW and .dat files; a .csv file, retrieved from the user history, that contains two columns (the .RAW filenames to be included in the analysis and a text label to indicate whether the file pertains to a biological sample or a ‘blank’) and an integer value representing the uniform number of replicates for each sample which is stored in the output as a common variable for use in subsequent tools. The LC-MS-specific part of the workflow, XCMS-Basic-Birmingham-Pipe, accepts a directory location as its first parameter. This tool will be described in more detail below.

To extract information from the .RAW files (i.e. mass spectra within the .RAW files or the meta data associated with transient data; see below), it is necessary to implement Microsoft Windows-specific dynamic link libraries (DLL), included as part of the 32/64 bit MSFileReader package (Thermo Scientific). Galaxy has been developed for deployment on Unix-based systems and therefore to achieve compatibility we have used the Windows emulator Wine [25] and custom tools written in Python [26] to read metadata from the .RAW files.

The preliminary stages of the DIMS processing act on a large number of files and produce a transformed but similarly large number of files. To facilitate passing of these multiple files from one module to another, and easier viewing of the workflow history, many tools make use of Galaxy’s ability to summarize output files in a single HTML file. File paths to the output files are provided as HTML links that are easily viewed within Galaxy and allow the user to interrogate and download the multiple outputs from the tool by simply clicking the link. At the same time, Galaxy tools can simply accept the solitary HTML file as an input and Galaxy facilitates interpretation of the multiple file locations stored within.

All tools in our workflow, with the exception of XCMS-Basic-Birmingham-Pipe, make use of .XML formatted files for passing common variables between one another. Initially these are produced by File List Manager and contain file names and other information as described above. An XML representation of the PLS-Toolbox (Eigenvector Research, Manson, USA) Dataset Object (DSO) has been used for storing the X matrix and associated metadata. This storage in human readable XML is intended to increase interoperability while maintaining the useful structure found in the DSO.

#### Workflow tools to process DIMS data (only)

**File List Manager:** collates file directory location, file name and blank/sample information for each .RAW file to be processed in an .XML file. Also records how many replicates there are for each biological sample.

**Sum Transients:** average each set of multiple transient data within each mass spectral SIM window (Note: this step is skipped when transient data is unavailable).

**Process Transients:** performs Hanning apodization, zero-filling, Fourier transformation and baseline correction to the averaged FT-ICR transient data, converting it from the time to frequency domain (Note: this step is skipped when transient data is unavailable).

**Mass Calibration and SIM-stitching:** picks peaks with a certain signal-to-noise-ratio threshold (e.g. 3.5:1), calibrates each SIM window to convert the frequency domain to *m/z* measurements and stitches all the SIM windows together to produce a peak list (of *m/z* values).

**Replicate Filtering:** filters peaks that fail to appear in at least x-out-of-n technical replicates (x chosen by user, *n* = number of technical replicates), thereby collating n technical replicates into a single spectrum; i.e. removes unreliable peaks.

**Align Samples:** aligns peaks across all samples.

#### Workflow tools to process LC-MS data (only)

**XCMS-Basic-Birmingham-Pipe:** Current implementation reads in the individual spectra (i.e. .mzML files) and groups/aligns spectral features across the samples using the R-package XCMS [23], returning a file with the X matrix represented as a comma separated value (.csv) file for ease of manipulation and two further .csv files, one with ‘row labels’, i.e. filenames, and one with ‘column labels’, i.e. *m/z* values.

From this point, the LC-MS workflow makes use of the tools developed for DIMS data and so the output is specifically configured to match the output of Align Samples. To perform this integration of workflows, it is necessary to also use the File List Manager tool to create the common .XML file containing basic file metadata.

#### Workflow tools to further process DIMS and/or LC-MS data

The first step in this part of the process joins the two workflows by converting their data to the DSO format. Subsequently all tools will expect data in the DSO format and will output an updated/transformed DSO.

**Create DSO:** combines the X data matrix file with row and column label information and class labels describing whether each sample is biological or blank. This data is stored as a DSO as used by PLS-Toolbox. This data structure is designed to hold information important for metabolomics style studies, e.g. data matrix, class information, axis scales, etc., and the PLS-Toolbox provides easy access to a suite of algorithms that are again, highly useful for statistical analyses and data visualization of multi-dimensional datasets.

**Blank Filtering:** compares peaks in biological samples to those appearing in any ‘blank’ samples and removes any that appear to be as strong in the blanks as in the biological spectra based on user-defined thresholds.

**Sample Filtering:** removes peaks that fail to appear in x-out-of-n samples (x chosen by user, *n* = number of biological samples in total or in any sample class), i.e. removes unreliable peaks.

#### Tools to manipulate the DSO structure

**Set Include:** defines the ‘include’ flag variable for either rows (samples) or columns (*m/z*) so that data may be removed from analyses without deleting it.

**Add Class List:** appends a list of sample groupings or classifications. The preceding Create DSO tool pre-populates a class list using sample/blank information provided to the File List Manager tool. This auxiliary tool allows alternative classification information to be added.

**Get Class List:** extracts either the text labels or integer representation of any class list in the DSO. This is expected to be useful for interoperability with other tools.

**Get Peak List:** extracts the *m/z* values and average peak intensities from the DSO, returning a tab delimited file. This is intended primarily for use with the MI-Pack software [10].

**Get X Matrix:** extracts the data (X) matrix as a .csv file. This format can be read easily by mainstream spreadsheet software e.g. Microsoft Excel, and can also be routinely handled by statistical software such as R.

**Get Axis Scale:** extracts the values which are stored in the ‘axis scale’ variable of the DSO. If the second axis dimension is chosen, this would represent the *m/z* values in a mass spectrometry DSO; the first dimension could be a continuous variable used as a regression factor.

#### Tools to prepare the X matrix for statistical analyses

At this stage the X data matrix requires preparation for statistical analysis, with the steps varying dependent upon whether uni- or multivariate analysis is to be performed. Our current Galaxy toolshed only includes multivariate analysis; thus all tools are required and it is strongly recommended to apply them in the following order:

**PQN Normalization:** applies Probabilistic Quotient Normalization to the sample filtered DSO [27].

**Missing Values Imputation:** imputes missing values using a KNN algorithm as described in Hrydziuszko and Viant [28].

***G*-log Transformation:** (only to be used for multivariate analysis) applies the generalized logarithm transform as described in Parsons and Viant [29] To stabilize the technical variance across all peaks, i.e., reduce the dominance of large, highly variable signals. To reduce the technical variance, the transformation parameter is optimized using a pooled ‘quality control’ sample that should exhibit only analytical variation across repeated measurements. Note that those samples used for optimization should be indicated using the ‘include’ flag variable, modified using Set Include. The *G*-log tool then optimizes the transformation parameter using ‘included’ samples and then applies that transformation to all samples. Set Include should be used again before subsequent analyses if all samples are to be included.

#### Workflow tools to conduct statistical analysis on DIMS and/or LC-MS data

**Principal Component Analysis (PCA) with Scores Test**: a script that applies PCA to the dataset. If the number of components is not specified, the tool first chooses the optimum number of components after interrogation of the cumulative variance in each component and then applies a univariate statistical test to the scores of each of the retained principal components (PCs) to produce a summary statistic for the degree of separation (along that PC) for each pair of classes. For the case of a two-class dataset a Student’s *t*-test is applied to this scores data, while an ANOVA followed by Tukey-Kramer *post hoc* pairwise comparison is applied in a multi-class study. There is no graphical output from this script; we believe that the subsequent statistical tests of separation are more reliable than visual interpretation. However, the model is saved and can be viewed graphically by the user outside the Galaxy environment.

#### Workflow tools to annotate DIMS and/or LC-MS data

MI-Pack [10] is a package written in Python developed for the interpretation and annotation of high-resolution mass spectra. Here, we have integrated three of the most widely used tools to allow the user to perform metabolite annotation.

**Empirical Formulae Search (EFS):** In our workflow, the first stage of putative metabolite annotation is to match the accurately determined masses (strictly speaking experimental *m/z* values) to one or more elemental compositions (C_c_H_h_N_n_O_o_P_p_S_s_) within a certain error tolerance.

**Single-Peak Search (SPS) and Transformation Mapping (TM):** each elemental composition and/or *m/z* value is searched against a specific chemical compound database(s) (e.g. KEGG, HDMB, LIPID MAPS) to assign a putative structure. Two approaches are implemented as described previously [10].

**Peak-Pattern Search (PPS):** extracts all the adduct patterns and relative isotopic abundance measurements from a peak list.

**Combine Outputs:** produces a summary file from single or combinations of different outputs (SQLite files) from the searching/mapping tools - effectively it allows easy viewing of the contents of the SQLite database files.

### Case studies

We have conducted two example workflows using small subsets of pre-published datasets from the MetaboLights repository. These rather trivial examples serve here simply to demonstrate all the steps involved in DIMS or LC-MS data processing. To aid reproducibility, these workflows are saved as two separate histories in the Galaxy distribution installed on the VM provided with this article [24].

#### Direct infusion mass spectrometry metabolomics

For depiction of the DIMS workflow, a subset of data was taken from the MetaboLights repository, accession MTBLS79. This data has previously been published and peer reviewed [21] and full details of the experimental conditions can be obtained from that publication. The subset is described in Additional file 1: Table S1 and was chosen to have two distinct classes of spectra (cow and sheep heart, 2 samples each), QC samples (2 samples) and a blank (1 sample). The DIMS workflow makes use of transient data (a series of .dat files) as well as .RAW format spectral file. These transient files were obtained from the original authors of the data as they are not included in the MetaboLights accession. The subset of data used here, including .dat files, is available from the GigaDB repository accompanying this publication both within the VM and separately [24].

**Fig. 2:**
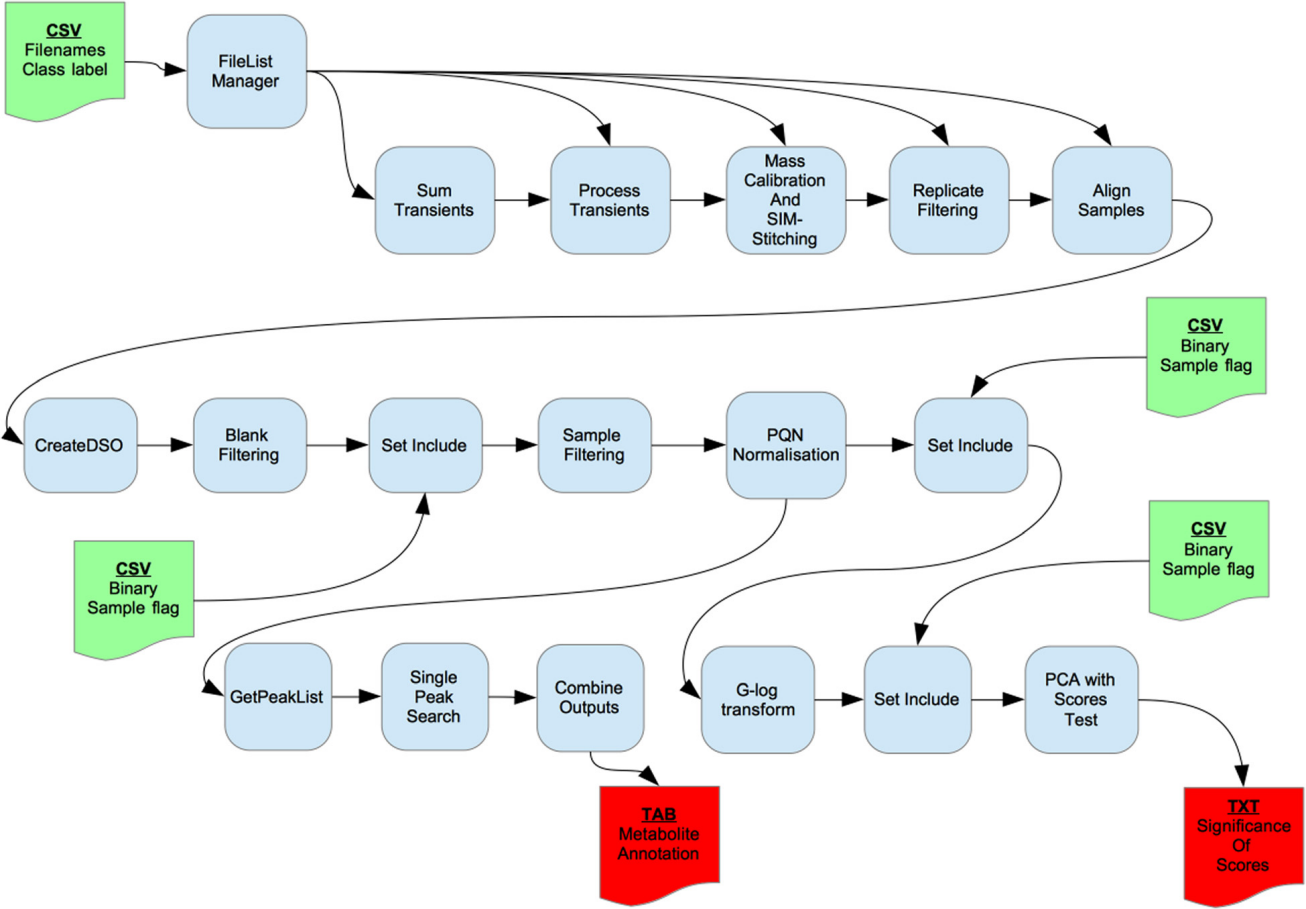
Workflow as applied to DIMS case study. Tools from the Galaxy workflow system are represented as round edged boxes; documents for input and output of configurations and results are square topped with curved bottoms (green for input, red for key output). Arrows indicate an intermediary output from one tool that is required as input by another later in the workflow

The workflow is depicted in Fig. 2. There are four uploaded inputs, all in .csv format. The first is a two column file providing filename and a label to indicate whether the file is a sample or blank, the other three files contain a binary list (comma separated) to indicate which samples should be included (1) or excluded (0) in subsequent steps. The first of these inclusion files removes the blank spectrum from the Sample Filter analysis. The second sets only the QC samples to be included before *G*-log transformation because the *G*-log parameters are optimized on the QC samples alone. The third sets only the biological samples to be included prior to PCA analysis, leaving a two-class comparison for the univariate test of PCA scores. Notably, the Sample Filter tool was run with a 100 % threshold meaning that there was no requirement to use the Missing Values Imputation tool. There are two main outputs, a text file containing a description of the PCA scores test and a tab-delimited file containing metabolite annotations produced by MI-Pack. The process to either endpoint is quite linear, with the exception of the initial File List Manager structure that is fed in to all SIM-stitch related tools.

The primary outputs from this workflow are a list of *p*-values associated with the significance of the separation of the two biological sample types (*t*-test, cow heart and sheep heart) on the first two PCs; see Additional file 1: Table S2. The metabolite annotations can be found in the Galaxy installation of the VM supporting this publication (published history ‘DIMS Test Data processing’).

#### Liquid chromatography-mass spectrometry

In the same manner as the DIMS example, a subset of LC-MS data was taken from an existing, published dataset within the MetaboLights repository, accession number MTBLS146 [30, 31]. This dataset was obtained from human maternal plasma at various stages of pregnancy; full details of the data collection can be found in the original research article. Additional file 1: Table S3 provides sample names and classifications for the test case, with the data being split between early stage pregnancy (13–16 weeks, 7 samples), late stage pregnancy (29–32 weeks, 7 samples) and QC samples (all stages of pregnancy, 5 samples). The subset of data used here is provided in the GigaDB repository accompanying this publication, both within the VM and separately [24, 32].

**Fig. 3:**
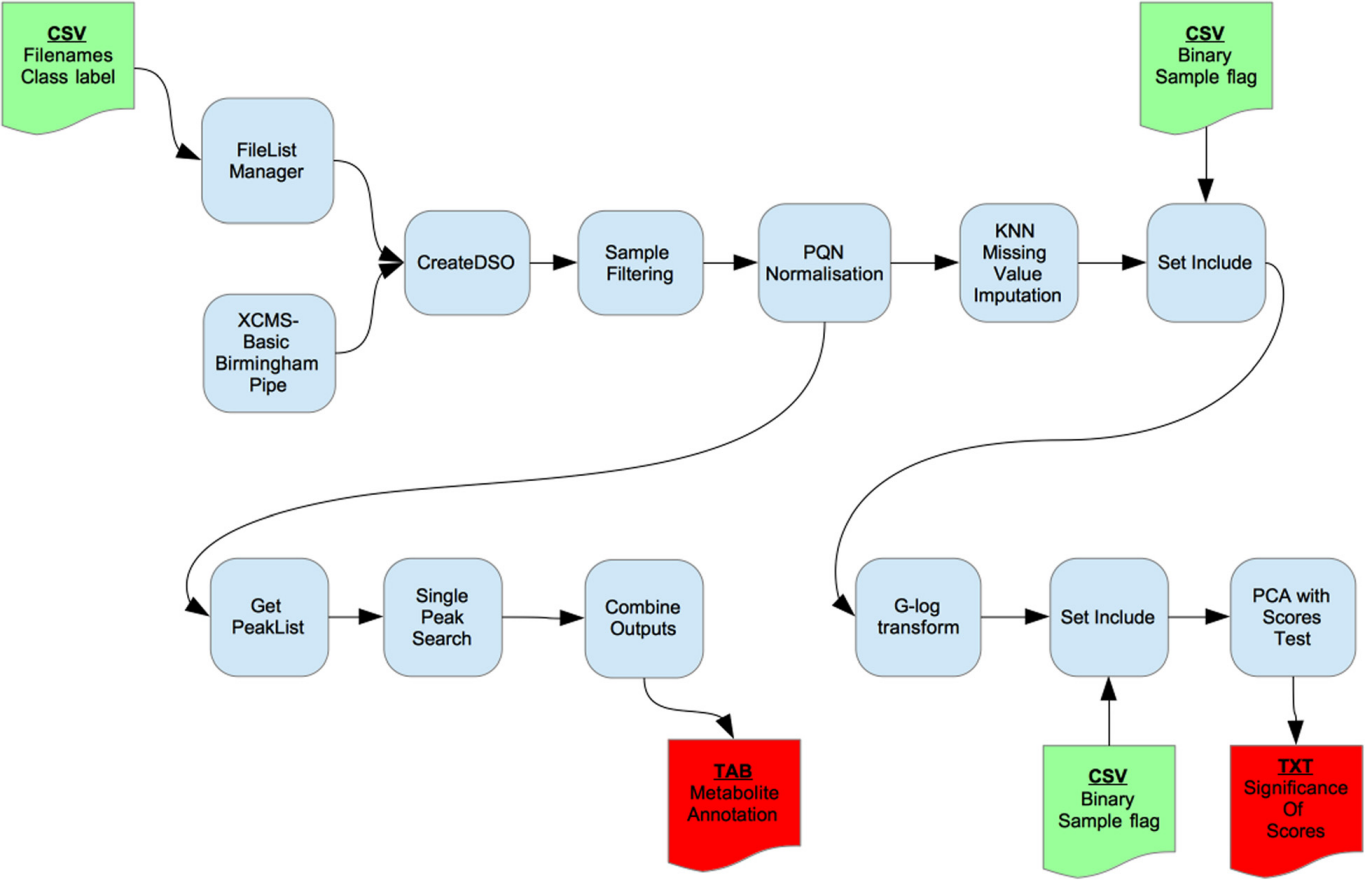
Workflow as applied to LC-MS case study. Tools from the Galaxy workflow system are represented as round edged boxes; documents for input and output of configurations and results are square topped with curved bottoms (green for input, red for key output). Arrows indicate an intermediary output from one tool that is required as input by another later in the workflow

Figure 3 presents the workflow diagrammatically. It can be seen to be similar to the DIMS workflow described above, except that the SIM-stitch code has been replaced with a single XCMS tool and there are no blank files, removing the need for the Blank Filter tool and the use of Set Include prior to Sample Filter. The three remaining input files are the same as described for the DIMS case study, the File List Manager tool is still used for initial recording of file information and the ‘inclusion’ files are used in the same manner for selecting QC samples prior to *G*-log and biological samples prior to PCA analysis. In this workflow, the Sample Filter tool was run with an 80 % threshold resulting in missing values and therefore the Missing Values Imputation tool was used.

Additional file 1: Table S4 provides the ‘PCA with Scores Test’ output for a two-class comparison between early and late stage pregnancy. Each PC contains a relatively small amount of variation and so seven PCs have been selected in total to produce a model that describes at least 70 % of the variance in the data. The results of the MI-Pack peak annotation of all peaks can be found in the Galaxy installation of the VM accompanying this paper (published history ‘LCMS Test Data processing’).

### International Galaxy metabolomics community

Here we have presented our initial implementation of DIMS and LC-MS workflows into the Galaxy environment. Although there is scope to expand our own local implementation, for example to include additional statistical tools or to introduce workflows to process NMR metabolomics spectra, it is arguably more valuable to begin to build an international Galaxy metabolomics community to share workflows. Specifically, our work complements the Galaxy workflows recently reported by the French Bioinformatics Institute and the French Metabolomics and Fluxomics Infrastructure (MetaboHUB; [13]), and those developed in the Netherlands [33] and the US [34]. In the near future, we anticipate a Galaxy toolshed [35] that will include a wide range of tools and workflows for processing and analyzing multiple types of metabolomics data, including more advanced statistical analyses. The tools reported here will be stored in the main Galaxy toolshed for easiest access by the Galaxy community. Beyond this, we seek to merge these workflows with other omics tools that have already been established in Galaxy’s large community-built repository. As the community continues to move towards public (and open access) repositories for the archiving of data, it will be important to integrate tools that use the industry standard file format for storing metadata, namely ISA-Tab [8], as used for example by the MetaboLights repository [8].

## Availability and requirements

**Project name:** Galaxy-M

**Project home page:** Viant-Metabolomics GitHub https://github.com/Viant-Metabolomics/Galaxy-M [36]

**Operating system(s):** UNIX (Galaxy); Platform independent for Galaxy’s browser-based user interface.

**Programming languages:** Python (version 2.7), Matlab Compiler Runtime (MCR) (version 8.3) or Matlab (version 2012a), PLS-Toolbox for multivariate tools (version 7.0.3) and R programming language (version 3.0.1, x86 64bit).

**Other requirements:** Galaxy [37], MI-Pack [10, 36], WineHQ (version 1.6.2, [25]), XCMS [23] and MSFileReader package (Thermo Scientific [38]).

**License:** GNU General Public License version 3.0 (GPLv3).

**Any restrictions to use by non-academics:** none.

**Virtual machine availability:** via GigaDB repository [24].

**Virtual machine accessibility:** Linux username = galaxym; Linux password = galaxym; Galaxy username = galaxym@galaxym.org; Galaxy password = galaxym; both case studies are available as published histories and published workflows in the Galaxy installation, or in the ‘galaxym’ user’s private history.

**Virtual machine system notes:** Ubuntu 14.04 LTS 64bit version (x86 architecture); graphical interface installed to allow easy access when stored locally; SSH port open (22).

## Availability of supporting data

Both datasets are available in full from the MetaboLights repository (MTBLS79 and MTBLS146), with the exception of the supporting .dat files for the DIMS data. The two subsets used here (including .dat files) can be found in the GigaDB repository for this paper [24] either in the VM or separately. Snapshots of the code and the virtual machine are also available from the GigaDB entry.

## Additional file

Additional file 1:
**Supplementary Tables 1-4.** (DOCX 23 kb)

## Abbreviations

DIMS: direct infusion mass spectrometry
DSO: dataset object
LC-MS: liquid chromatography mass spectrometry
PCA: principal component analysis
SIM: selected ion monitoring
VM: virtual machine
