# Supplementary material for: Galaxy-M: a Galaxy workflow for processing and analyzing direct infusion and liquid chromatography mass spectrometry-based metabolomics data
Source: Gigascience. 2016 Feb 23;5:10. doi: 10.1186/s13742-016-0115-8 (PMC4765054; doi:10.1186/s13742-016-0115-8)
Supplement: Additional file 1: — Supplementary Tables 1-4. (DOCX 23 kb) [file 13742_2016_115_MOESM1_ESM.docx]

# **Additional file 1 in support of “**Galaxy-M: A galaxy workflow for processing and analysing direct infusion and liquid chromatography mass spectrometry-based metabolomics data”

**Supplementary Table 1.** **Spectral data used in DIMS case study (MetaboLights accession, MTBLS79)**

| **Source** | **File Names (Thermo Xcalibur .RAW files)** | **Sample Name (MetaboLights)** |
| --- | --- | --- |
| Cow heart | batch01_C1_rep01_22.RAW (plus replicates _23.RAW and _24.RAW) | batch01_C01 |
| Cow heart | batch01_c10_rep01_25.RAW (plus replicates _26.RAW and _27.RAW) | batch01_C10 |
| Sheep heart | batch01_S7_rep01_19.RAW (plus replicates _20.RAW and _21.RAW) | batch01_S07 |
| Sheep heart | batch01_S1_rep01_31.RAW (plus replicates _32.RAW and _33.RAW) | batch01_S01 |
| QC | batch01_QC_rep01_04.RAW (plus replicates _05.RAW and _06.RAW) | batch01_QC02 |
| QC | batch01_QC_rep01_07.RAW (plus replicates _08.RAW and _09.RAW) | batch01_QC03 |
| blank | batch04_B_rep01_301.RAW (plus replicates _302.RAW and _303.RAW) | batch04_B02 |

Each sample has three replicates collected consecutively. The file naming convention indicates these consecutively collected replicates with the last characters of the filename indicating the ‘run order’ of the collection e.g. the 2nd replicate of a file named batch01_C1_rep01_22.RAW will be batch01_C1_rep02_23.RAW, which is abbreviated to _23.RAW. Note that each .RAW file is accompanied by several .DAT transient files that are not included in the MetaboLights repository. This data is available in the GigaDB repository for this publication, both in the VM and separately [24].

**Supplementary Table 2.** **Principal component analysis results from DIMS case study**

| **Principal component (PC)** | ***P-*value** | **Percentage of variance** |
| --- | --- | --- |
| 1 | 0.094 | 65% |
| 2 | 0.606 | 29% |

The p-values indicate the significance in separation of two classes (cow, sheep) when the scores for a particular principal component are analyzed with a t-test. No correction for multiple testing is made.

**Supplementary Table 3.** **Data used in LC-MS case study (MetaboLights accession, MTBLS146)**

| **Source: Pregnancy maternal plasma** | **File Names (*.mzML)** | **Sample Name (MetaboLights)** |
| --- | --- | --- |
| 13-16 weeks | AR02407_1 | B04 |
|  | AR02412_1 | B05 |
|  | AR02422_1 | B06 |
|  | AR02435_1 | B07 |
|  | AR02500_1 | B10 |
|  | AR02554_1 | B15 |
|  | AR02723_1 | B25 |
| 29-32 weeks | AE00291_1 | F01 |
|  | AJ02668_1 | F03 |
|  | AJ02988_1 | F06 |
|  | AR02624_1 | F09 |
|  | AR03026_1 | F07 |
|  | BD00005_1 | F17 |
|  | BC00410_1 | F14 |
| QC (9-32 weeks) | QC10_1 | QC |
|  | QC6_1 | QC |
|  | QC7_1 | QC |
|  | QC8_1 | QC |
|  | QC9_1 | QC |

The file names and associated sample names from the MetaboLights repository are listed to allow the user to find the data in the larger repository. This subset of data is supplied in the GigaDB repository accompanying this publication both in the VM and separately[24,32].

**Supplementary Table 4.** **Principal component analysis results from LC-MS case study**

| **Principal component (PC)** | **p-value** | **Percentage of variance** |
| --- | --- | --- |
| 1 | 0.493 | 17% |
| 2 | 0.085 | 11% |
| 3 | 0.136 | 11% |
| 4 | 0.023 | 10% |
| 5 | 0.947 | 8% |
| 6 | 0.755 | 8% |
| 7 | 0.977 | 7% |

The p-values indicate the significance in separation of two classes (early pregnancy: 13-16 weeks, late pregnancy: 29-32 weeks) when the scores for a particular principal component are analyzed with a t-test. No correction for multiple testing is made.
